# Effect of multiparametric MRI and ultrasound-guided biopsy for early prostate cancer detection: A prospective cohort study

**DOI:** 10.6026/973206300214015

**Published:** 2025-11-15

**Authors:** Deepika Karunakaran, Holebasu Ballur, Archana Abarnadevi Karthikeyan, Satya Sudhakara Bhat, Kausalya Perumalsamy, Anannya Vaibhavi

**Affiliations:** 1 Department of Emergency Medicine, University Hospitals of Morecambe Bay, NHS Trust, United Kingdom; 2 Department of Radio-Diagnosis, KLE Jagadguru Gangadhar Mahaswamigalu Moorsavirmath Medical College, Hubli, Karnataka, India; 3 Department of HPB Surgery, Kings College Hospital, London, England, United Kingdom; 4 Department of Trauma and Orthopaedics, Lancashire Teaching Hospitals NHS Foundation Trust, United Kingdom; 5 Department of Pediatrics, Resident Medicla Officer, Kauvery Hospital, Tirunelveli, Tamil Nadu, India; 6 Department of Physiology, RML hospital, Lucknow, Uttar Pradesh, India

**Keywords:** Multiparametric MRI, USG biopsy, prostate cancer, magnetic resonance imaging

## Abstract

It is essential to detect clinically significant prostate cancer (csPCa) in an early and precise manner. However, there are challenges given that the usual TRUS-guided biopsy may leave tumors undiagnosed, or only detect indolent disease. In this prospective cohort study of 200 patients, we compared the diagnostic accuracy of multiparametric MRI (mpMRI) with TRUS-guided biopsy. The study found better sensitivity (91% vs 68%) and specificity (76% vs 60%) than TRUS biopsy, and identified 22% more clinically significant cancers than TRUS biopsies. In addition, mpMRI showed better positive predictive value and negative predictive value than TRUS-guided biopsy; this suggests general advantage in diagnostic accuracy and performance for mpMRI. Data shows, there is support for mpMRI having improved efficacy as a method of detecting clinically significant prostate cancer early compared to TRUS-guided biopsy.

## Background:

Prostate cancer is among the most common cancers in men globally and represents a significant cause of morbidity and mortality related to cancer [1]. The worldwide burden of prostate cancer continues to increase, especially in aging populations. A central clinical dilemma in prostate cancer lies in identifying clinically significant prostate cancer (csPCa) prostate cancer that needs intervention from indolent tumors that may remain asymptomatic and do not require treatment [2]. Overdiagnosis and overtreatment remain a large concern, which highlights the need for sound diagnostic strategies that can effectively detect csPCA while minimizing unnecessary intervention [3]. Historically, transrectal ultrasound (TRUS)-guided biopsy has been the historical standard for diagnosis of prostate cancer. Although they are accessible and relatively cost-effective, TRUS-guided biopsies are associated with a number of disadvantages, including inadequate sampling of certain regions (particularly the anterior and apical zone of the prostate) and detecting clinically insignificant cancers that do not impact survival [4]. The random, systematic-sampling associated with TRUS-guided biopsies can result in false negatives and overdiagnosis, both of which improve the overall efficiency of the diagnostic [5]. Multiparametric magnetic resonance imaging (mpMRI) has become a popular tool in prostate cancer diagnosis and characterization in recent years. MpMRI uses multiple imaging sequences including T2-weighted imaging for anatomic detail, diffusion-weighted imaging (DWI) to identify cellular density, and dynamic contrast-enhanced (DCE) imaging for vascular characterization, ultimately increasing both sensitivity and specificity in detecting suspicious lesions [6]. The introduction of the Prostate Imaging-Reporting and Data System (PI-RADS) has also reduced variability and helped standardize mpMRI reports, improving results across institutions [7]. Clinical evidence increasingly supports the mpMRI role in prostate cancer diagnostics. The PRECISION trial is a landmark multi-center study that demonstrated mpMRI with targeted biopsy resulted in a higher diagnosis of csPCa, and reduced the diagnosis of clinically insignificant tumors, compared with standard TRUS-guided biopsy [8]. Confirmatory evidence from meta-analyses and prospective studies show mpMRI is superior in the detection of cancer compared with TRUS biopsy, and mpMRI can also avoid spare biopsies in patients when mpMRI is negative, which reduces complications (such as infection, bleeding, and discomfort) , and is a great benefit [9]. Unfortunately, cost, access to quality community imaging, and radiological expertise to correctly interpret mpMRI, still hamper widespread mpMRI use in practice, despite such benefits [10]. Therefore, it is of interest to compare the diagnostic performance of multiparametric MRI with conventional TRUS-guided biopsy in the early detection of clinically significant prostate cancer.

## Aim:

To assess and compare the diagnostic performance of multiparametric magnetic resonance imaging (mpMRI) and standard transrectal ultrasound (TRUS)-guided biopsy in the diagnosis of clinically significant prostate cancer in patients with elevated prostate-specific antigen (PSA) levels and/or abnormal digital rectal examination (DRE).

## Objectives:

## Primary objective:

To compare the detection rates of clinically significant prostate cancer (csPCa), defined as Gleason score ≥ 3+4, between mpMRI-targeted biopsy and TRUS-guided biopsy.

## Secondary objectives:

[1] To assess and compare the sensitivity, specificity, positive predictive value (PPV), and negative predictive value (NPV) of mpMRI and TRUS-guided biopsy in detecting csPCa.

[2] To evaluate the correlation between PI-RADS scores on mpMRI and histopathological outcomes.

[3] To identify cases of clinically significant prostate cancer detected exclusively by mpMRI-targeted biopsy and missed by TRUS-guided biopsy.

[4] To analyse concordance between mpMRI findings and histopathological diagnosis using kappa statistics.

[5] To determine the potential of mpMRI as a first-line diagnostic tool in patients with suspected prostate cancer.

## Materials and Methods:

This research aimed to carry out a prospective cohort study at an academic urology center from January 2024 to December 2024. Eligible men aged 50-75 years had a prostate-specific antigen (PSA) of ≥ 4 ng/mL and suspicious findings on digital rectal examination (DRE) and had not been previously diagnosed with prostate cancer. Excluded patients had previous prostate biopsy, contraindications to MRI i.e. pacemakers or metal implants, or had an active urinary tract infection. All patients underwent multiparametric magnetic resonance imaging (mpMRI) with a 3 Tesla MRI without the use of an endorectal coil. The images were read according to the PI-RADS version 2.1 criteria. All patients underwent a systematic 12-core transrectal ultrasound (TRUS) guided biopsy. If mpMRI images captured lesions that were scored ≥PI-RADS 3 they were also targeted to obtain biopsies. The main goal of the investigation was the detection rates of clinically significant prostate cancer (defined as Gleason score ≥3+4), while secondary goals were detection rates of any prostate cancer, and the sensitivity, specificity, positive predictive value (PPV), and negative predictive value (NPV) of mpMRI in relation to cancer detection. Descriptive statistics were used to summarize the data and McNemar's test was used to provide paired comparisons of detection rates across systematic and targeted biopsy approaches.

## Statistical significance:

[1] The difference in detection rates was statistically significant (p < 0.001)

[2] Concordance between mpMRI and pathology was high (κ = 0.79)

## Results:

A total of 200 patients were included in the study, with a mean age of 64.3 ± 6.2 years and a median PSA level of 6.7 ng/mL (Table 1 - see PDF). Multiparametric MRI (mpMRI) revealed PI-RADS ≥3 lesions in 148 patients (74%), and 110 patients (55%) were histologically confirmed to have clinically significant prostate cancer (csPCa, defined as Gleason score ≥3+4) (Table 1 - see PDF). Regarding diagnostic performance, mpMRI demonstrated superior sensitivity (91%) and specificity (76%) compared to transrectal ultrasound (TRUS)-guided biopsy, which had 68% sensitivity and 60% specificity (Table 2 - see PDF). The positive predictive value (PPV) and negative predictive value (NPV) of mpMRI were 84% and 88%, respectively, whereas TRUS-guided biopsy showed PPV of 72% and NPV of 63% (Table 2 - see PDF). MpMRI identified 101 of 110 clinically significant cases (91.8%), while TRUS-guided biopsy detected only 75 cases (68.2%). Notably, 12 patients were diagnosed exclusively through mpMRI-targeted biopsy, which were missed by systematic TRUS-guided biopsy, a difference that was statistically significant (p < 0.001). Substantial agreement between mpMRI findings and histopathology was observed, with a kappa coefficient (κ) of 0.79. These results confirm that mpMRI offers superior sensitivity, specificity, and lesion localization compared to TRUS-guided biopsy, allowing for more accurate detection of clinically significant prostate cancer while reducing overdiagnosis of low-grade tumors. The within-subject comparison strengthens these findings, although the study is limited by its single-center design and absence of long-term outcome data.

Table 1 (see PDF) shows that the study cohort (n=200) had a mean age of 64.3 years and a median PSA of 6.7 ng/mL, with 74% of patients demonstrating PI-RADS ≥3 lesions on mpMRI and 55% confirmed to have clinically significant prostate cancer (csPCa). Table 2 (see PDF) shows that mpMRI had higher diagnostic accuracy than TRUS-guided biopsy, with sensitivity of 91% versus 68%, specificity of 76% versus 60%, and superior positive and negative predictive values, indicating more reliable identification of csPCa. Table 3 (see PDF) shows that mpMRI-targeted biopsy detected 101 of 110 csPCa cases (91.8%), including 12 cases missed by TRUS-guided biopsy, which detected only 75 cases (68.2%), highlighting the incremental benefit of mpMRI. Table 4 (see PDF) shows that higher PI-RADS scores strongly correlated with clinically significant lesions, especially in the peripheral zone, demonstrating mpMRI's ability to accurately localize and characterize high-risk prostate lesions. Table 5 (see PDF) shows substantial agreement between mpMRI findings and histopathology, with a kappa coefficient of 0.79, supporting the reliability of mpMRI for targeted biopsy planning. Table 6 shows that mpMRI consistently outperformed TRUS-guided biopsy across all PSA subgroups (<4, 4-10, >10 ng/mL), particularly in lower PSA ranges, demonstrating robust detection even in patients with traditionally low-to-intermediate risk.

## Discussion:

This prospective cohort study shows that multiparametric magnetic resonance imaging (mpMRI) has better sensitivity and specificity for the early diagnosis of clinically significant prostate cancer than traditional transrectal ultrasound (TRUS)-guided biopsy [11]. mpMRI's ability to localize and characterize prostate lesions allows for targeted biopsies which maximize the detection of clinically significant tumors while minimizing the detection of indolent, low-grade cancers which may not require immediate treatment [12]. This specificity also minimizes the risk of overtreatment, an important concern in prostate cancer. The aims of this study align with recent large-scale trials demonstrating that an mpMRI-first diagnostic approach improves the accuracy of prostate cancer detection and reduces the number of unnecessary biopsy procedures [13]. mpMRI allows for biopsy targets to be selected by the identification of suspicious areas using high-resolution image quality with functional sequences [14]. Imaging allows clinicians to select biopsy targets to improve yield and reduce the amount of sampling error [15]. In comparison, TRUS-guided biopsy is systematic but still virtually random sampling, which may miss anterior or small lesions resulting in high false negative rates of clinically significant disease [16]. One noteworthy advantage of this research study is the within-subjects design where every patient had an mpMRI-targeted biopsy and a TRUS-guided biopsy [17]. By having the same patient undergo both types of biopsies it allows for a direct, head-to-head comparison of the two modalities in the same anatomical environment, reducing inter-patient variability, and allowing for more precise evaluation of diagnosis performance compared to the other [18]. In addition, the imaging protocols and biopsy procedures were standardized to reduce variability in data collection and interpretation, thus strengthening the validity of results. In spite of these advantages, some limitations must be considered. This was a single center study so the results may not generalizable to other hospitals with different levels of expertise or imaging capacity [19]. The follow-up time was also limited, precluding assessment of long-term outcomes like disease progression, biochemical recurrence or treatment-related outcomes. As patients appearing for biopsy may be at a different risk profile than the general population being screened for prostate cancer, there may also be an inherent selection bias in the study population [20]. The potential relevance to practice from this study is and will continue to be an important one. Using mpMRI has the potential to improve patient stratification and enhance targeted biopsy diagnostic efficiency to lesions most likely to be clinically significant cancers. This, in turn, will help to prevent patient suffering and morbidity as a result of unwarranted biopsies with the associated probability of having pain, bleeding, infection, or anxiety in patients. Importantly, where resources are limited, mpMRI may also help in optimally assigning biopsy procedures to patients with a higher pre-test probability of disease. Future work will be needed to validate these findings in larger, multi-center trials across different populations and practices. Longitudinal studies on oncological outcomes and cost-effectiveness will also be needed to determine the effect of mpMRI pathways have on patient care more generally. Finally, new, future technologies, including artificial intelligence supported mpMRI appraisal and fusion-based systems, will help provide new avenues for increased mpMRI diagnostic accuracy, lower operator bias, and ease of implementation into the clinical workflow.

## Conclusion:

Multiparametric MRI outperforms TRUS-guided biopsy in detecting clinically significant prostate cancer and shows promise as a primary diagnostic tool. Incorporating mpMRI into the diagnostic pathway can improve early detection and reduce unnecessary interventions.
